# Self-enucleation of the right eye by a 38-year-old woman diagnosed with schizoaffective disorder: a case report

**DOI:** 10.1186/s12888-020-02974-6

**Published:** 2020-11-25

**Authors:** Natalia Chechko, Eva Stormanns, Klaus Podoll, Susanne Stickel, Irene Neuner

**Affiliations:** 1 Department of Psychiatry, Psychotherapy and Psychosomatics, Medical Faculty, Uniklinik RWTH Aachen, Pauwelsstrasse 23, 52074 Aachen, Germany; 2 Institute of Neuroscience and Medicine: JARA-Institute Brain Structure Function Relationship (INM 10), Research Center Jülich, Jülich, Germany; 3 Institute of Neuroscience and Medicine (INM-7), Forschungszentrum Juelich, Juelich, Germany; 4 Institute of Neuroscience and Medicine (INM-4), Forschungszentrum Juelich, Juelich, Germany

**Keywords:** Self-enucleation, Schizoaffective disorder, Delusion of sin, Self-mutilation

## Abstract

**Background:**

Autoenucleation is a rare form of self-mutilation typically associated with psychiatric disorders such as schizophrenia, substance-induced psychosis and bipolar disorder. The act is usually unilateral, although bilateral attempts are also well documented in the literature.

**Case presentation:**

It is a case study involving a female patient (NN) diagnosed with schizoaffective disorder who self-enucleated her right eye following sexual intercourse with a fellow patient, and was forcefully prevented by staff from enucleating the second eye. We report recurrent episodes of her illness culminating in this severe act of self-mutilation. The motivational reasons behind this form of self-harm along with differential diagnosis and potential treatment options are discussed in the context of the available literature.

**Conclusion:**

Autoenucleation is commonly associated with religious and sexual delusions, and patients are thought to be at a greater risk of further self-harm. Timely antipsychotic treatment is likely to reduce the risk of such extreme forms of self-harm, although they can occur despite robust therapeutic intervention and treatment attempts. While self-inflicted eye injuries are rare, their prevention in what is typically a difficult patient group is fraught with challenges.

## Introduction

“If your right eye causes you to sin, tear it out and throw it away.

For it is better that you lose one of your members than that your whole body be thrown into hell.”

Mathew 5:29

The act recommended in the Bible verse above is self-enucleation, also known as autoenucleation, which is a self-inflicted, complete removal of one’s eye [1].

With an average of 1 in 30 million people, autoenucleation is a rare form of self-mutilation typically associated with psychiatric disorders such as schizophrenia, substance-induced psychosis and bipolar disorder [1–3]. It has also been seen, though more rarely, among patients with obsessive-compulsive disorder, depression as well as mental retardation, neurosyphilis, Lesch-Nyhan syndrome or structural brain lesions [3, 4]. The attempt is usually unilateral, although bilateral attempts are also well documented in the literature [3]. While hypersexual or hyper-religious delusions have often been seen as reasons behind this form of self-mutilation, the motivations seem to vary and frequently ensue from psychotic conditions [3, 5, 6].

## Case report

### Psychiatric history

NN had been diagnosed with severe depression for the first time around 2002/2003, when she was in her early twenties. Although she was also described as delusional, with ideas of reference and persecution, the initial diagnosis was depression and treatment with amisulpride stabilized her.

Two hypomanic phases over the next few years led NN to being diagnosed with bipolar disorder. After a relatively stable period of about 7 years, she experienced a recurrence of depressive episodes and was treated with citalopram in addition to amisulpiride, which kept her symptoms in check for approximately 8 years. In 2018, due to complaints about anxiety and restlessness in relation to the citalopram treatment, the medication was changed to quetiapine for its mood stabilizing effect.

NN also suffered from Hashimoto’s thyroiditis and obesity.

After finishing school, NN had completed an apprenticeship in healthcare and worked as a nurse in a psychiatric hospital’s intensive care unit for several years. While working as a nurse, she also pursued a bachelor’s degree in social work, obtaining the degree in 6 years. In 2017, she started working as a social worker supporting people with psychiatric disorders. Her academic and professional achievements were made possible by the relatively long periods of stability in her psychiatric history.

### Current episode

In early 2019, she came, on her own, to the emergency department of University Hospital Aachen, reporting that she had been signed off work for 3 weeks because of a depressive episode. She also reported that her medication with amisulprid had been interrupted for a while and then started again as she felt more depressed following its discontinuation. Despite the resumption of her medication, she reported a recent worsening of her condition, causing her to ruminate, wake up earlier than usual, and feel an inner unrest with chastened mood. The neurological examination, along with electroencephalography, electrocardiogram and the conventional magnetic resonance imaging, showed no pathological changes. After 2 days in the secure psychiatric unit, NN was moved to the open ward specialized in treating psychotic patients. While in the earliest phase of the treatment she showed only depressive symptoms, over time she was found to be increasingly unstable and suicidal. On several occasions, she needed to be admitted to the secure unit for stays ranging from 2 days to several weeks. About 2 months into her stay at the hospital, she reported suicidal thoughts while on a trip with fellow patients and an occupational therapist from the ward. She walked on the road instead of the sidewalk, and later explained that she was hoping to get run over by a car because she could no longer tolerate her circumstances. As she continued to express suicidal thoughts without the assurance that she would not attempt self-harm, she was moved to the secure unit for several days.

In the later period of her stay at the hospital, NN become increasingly psychotic. In the weeks leading up to her autoenucleation, she was persistently delusional, stating she did not trust anyone and that everyone in the city knew she was a psychiatric patient and had seen videos of her being naked. She was almost constantly agitated, often requesting conversations and contact with the staff. She expressed feelings of desperation and guilt and feared that her stay at the hospital might not be paid for.

### Course of treatment

The quetiapine monotherapy dose was raised first to 500 mg and then, 2 weeks before her autoenucleation, up to 800 mg. Despite the elevated plasma levels of quetiapine seen during therapeutic drug monitoring, no improvement with respect to her psychotic symptoms was detected. Previously, a combination therapy with quetiapine and amisulpride and later with quetiapine and aripiprazole as well as with olanzapine had also been applied without any significant effect.

Because of the foreground depressive symptoms, she was additionally treated with the antidepressive agent sertraline (up to 150 mg), which was changed, due to persistent unrest and anxiety, first to agomelatine (25 mg) and then to bupropion (up to 300 mg), which was later substituted with duloxetine (up to 90 mg). NN reported that no antidepressant therapy improved her mood. She was also treated with benzodiazepines but did not consent to lithium medication.

Besides medication, the patient tried to use certain relaxation techniques she had learned to help reduce stress, but for the most part they were not of much help. Throughout her stay at our hospital, NN received psychotherapeutic support on a regular basis.

Neuropsychological assessment shortly after the initial admission to the hospital: According to the neuropsychological assessment, NN had an average verbal intellectual capacity (IQ 92; Test for Verbal Intelligence) [7] and an intact processing speed (Trail-Making-Test-A [TMT-A]) [8]. The executive functions in terms of cognitive flexibility (TMT-B) [8] and divergent problem-solving ability (test for verbal fluency) [9] were found to be impaired. While short-term and working memory (WMS-R) [10] were preserved, logical memory (WMS-IV) [11] was seen to be compromised. The Minnesota Multiphasic Personality Inventory (MMPI-2; Self-assessment scale) [12] showed a striking personality profile with elevated scores on the scales for hypochondria, depression, psychopathy, paranoia, schizophrenia, psychasthenia and hypomania. On another self-assessment scale, NN reported severe depressive symptoms (BDI = 36, Beck Depression Inventory; BDI-II) [13].

### Autoenucleation

The incident occurred on the day she was found having sex in a bathroom with a fellow male patient. She later admitted to having enticed the male patient by working out on the ward’s cross trainer and masturbating in front of him. She described the motivation behind her actions as self-punishment, adding that she felt unclean and guilty throughout the experience. The nursing staff had interrupted the sexual intercourse. NN then went to the common room and took off her clothes once again. The patient who had had sex with NN sounded the alarm about NN trying to pull out her eye. By the time the nursing staff arrived, NN had already enucleated her right eye, which was lying on the floor next to her, and was attempting to pull out the other eye. She was naked and covered in blood. The staff had to use force to prevent her from enucleating the second eye. While the staff was immobilizing her, she repeatedly said that the other eye had to be enucleated as well. She also mentioned that she thought she was the reincarnation of Adolph Hitler. When the doctors in charge of psychiatry and ophthalmology arrived, NN seemed quiet and cooperative. Asked if she felt any pain, she said that she did not and allowed the doctors to examine her. After the ophthalmologic examinations were done and she was back in her room (immobilized), she told the psychiatric doctor in charge that she had not heard any voices commanding her to remove her eye. Instead, she said, that it was her overwhelming sense of guilt and self-hatred that had prompted her to initiate the sexual act, which was self-punishment; she wanted to debase herself. And after the sexual act, she felt worse with the tension inside her rising even higher. She denied ever having thought about removing her eyes before. Thus, based on the patient’s account, the autoenucleation happened spontaneously without premeditation. While reflecting on the incident, she appeared fully oriented, denying any delusional thoughts (or voices heard) to be responsible for her action and identifying, instead, her sense of tension, guilt and self-hatred as the trigger.

### Treatment following the autoenuclaetion

After the incident, the dosing of the antipsychotic medication was raised once again. The best antipsychotic and mood stabilizing response was achieved with a combination of quetiapine (800 mg/d), risperidone (9 mg/d), haloperidol (5 mg) and valproate (risen up 1,8 g/d). In addition, she was briefly treated anxiolytically with lorazepam (up to 5,5 mg/d). However, the latent psychotic ideas persisted and appeared to diminish only after multiple electroconvulsive therapy treatments, following which haloperidol was discontinued without any psychotic relapse. Even after being discharged from the hospital 6 months later, the patient was unable to return to normal life and was, therefore, sent to a care home for the mentally ill.

## Discussion and conclusion

The case involving NN was atypical for multiple reasons, including the absence of the characteristics of disorganization, good premorbid functioning, resistance to antidepressant and antipsychotic treatment, inner tension and sensations of guilt and self-hatred as purported motivations for autoenucleation, and the lack of any obvious trigger for the psychiatric relapse after a long stable period. These factors made the case a particularly challenging one with respect to diagnosis as well as a clear understanding of the patient’s motivation. Although several possible conditions (depression with psychotic symptoms, bipolar affective disorder with psychotic symptoms, borderline personality disorder, schizophrenia and anti-NMDA receptor encephalitis) were taken into account by way of differential diagnosis, NN was finally diagnosed with schizoaffective disorder (SZD) as her illness had an uninterrupted duration (criterion A of DSM-5 for SZD [14]) with a major depressive episode in addition to meeting the criterion for schizophrenia. While the predominant psychotic symptom was delusion, symptoms meeting the criterion for a major mood episode (criterion C) were also present throughout the patient’s stay at the hospital as well as during the residual phase of her illness following discharge. However, it was unclear whether NN’s case met criterion B, which requires that delusions or hallucinations be present for 2 weeks or more in the absence of a depressive or manic episode during the course of the illness, because the patient was unable to provide any relevant information during the cross-sectional assessments. In spite of this uncertainty, the severity of NN’s depressive episode, the hypomanic history and the psychotically motivated action of autoenucleation made SZD the most appropriate diagnosis. However, in certain important respects the case was atypical, calling for the consideration of an additional differential diagnosis. NN’s high levels of functional ability sustained over a long period following the first psychotic and depressive episode (she had successfully worked as a nurse and a social worker) and her cognitive abilities were particularly noteworthy. During conversations, she mostly seemed properly orientated and capable of abstract reasoning. The lack of disorganization and her robust premorbid functioning are atypical for SZD, providing the basis for schizophrenia being completely ruled out and a bipolar disorder with psychotic symptoms being considered as the closest differential diagnosis. On the other hand, the nature of her psychotic symptoms (transitory belief to be the reincarnation of Adolph Hitler, paranoia, and delusion) appeared to be more typical of SZD compared to bipolar disorder. The diagnosis of depression with psychotic symptoms was ruled out based on the patient’s history of hypomanic episodes.

As regards the motivation behind autoenucleation, NN stated that she performed the act because she also could not tolerate the inner tension and the sensations of guilt and self-hatred. Based on her mood swings, suicidal behavior and inner tension, a borderline personality disorder (BPD) was considered as the potential underlying disease, taking the psychotic symptoms into account in the context of transient stress-related paranoid ideation. But eventually BPD was deemed implausible given the mix of stable and unstable phases in NN’s life. As she began to recover from her psychosis, she reflected on her self-mutilation, expressing regret at the loss of an eye and her gratitude to the doctors and the nursing staff for preventing her from losing the other eye. This, coupled with the lack of any further attempts at self-mutilation following recovery, did not support the diagnosis of BPD. Nevertheless, apart from the symptoms of SZD, the patient showed dependent personality traits and was emotionally unstable. In addition, the fact that her symptoms remained quite therapy-resistant with respect to antidepressant and antipsychotic treatment raises questions as to the extent to which personality traits might have contributed to the progress of her illness. However, her response to electroconvulsive therapy indicates a biological vulnerability to depression and psychosis. In addition, the inflammatory nature of the psychosis (e.g. anti-NMDAR encephalitis) cannot be completely ruled out as NN refused to undergo lumbar puncture. However, a psychotic episode in the past, no difficulties with respect to orientation or concentration, well-preserved short-term and working memory and no neurological deficits shown in conventional magnetic resonance imaging constitute a persuasive argument against any inflammatory causation.

According to the psychiatric literature on autoenucleation, it is commonly associated with religious and sexual delusions [3, 15–17]. These patients often refer to concepts of sin, evil, guilt and atonement as their motives for self-harm. A similar pattern was observed in NN with her act of autoenucleation having been provoked by sex with another patient. She reported feeling debased and guilty, citing self-punishment as the motivation behind her sexual actions. That she undressed herself before the enucleation indicates an experience of sin. Also, her feeling, at the moment of the autoenucleation, that she was the reincarnation of Hitler points to the perception of evil. According to the medical and psychiatric literature, autoenucleation patients are at a greater risk of further self-harm [3]. In our patient’s case, it was only the intervention of doctors and nurses that protected her from a second self-enucleation. Remarkably, however, NN reported feeling no physical pain during the self-mutilation, confirming the assumption that self-mutilators may have a higher pain threshold [4, 18].

Self-mutilation of this severity is so rare that it cannot be predicted accurately unless there is a history of attempts at self-harm or overt expressions of such desire [5, 6]. Timely antipsychotic treatment is likely to reduce the risk of such extreme forms of self-harm, but, as our case report demonstrates, they can occur despite robust therapeutic intervention and treatment attempts. Although self-inflicted eye injuries are rare, their prevention in what is typically a difficult patient group is fraught with challenges.

## Data Availability

Data sharing is not applicable to this article as no datasets were generated or analyzed during the study. Identifying/confidential patient data cannot be shared.

## Abbreviations

SZD: Schizoaffective disorder
BPD: Borderline personality disorder
